# On-site detection of fish furunculosis by combining DNAzyme and carboxyl-functionalized graphene

**DOI:** 10.3389/fchem.2022.1015827

**Published:** 2022-09-08

**Authors:** Wen Ding, Qingzhen Miao, Xiuli Bao, Shiyuan Wang, Jing Lu, Mingsheng Lyu, Shujun Wang

**Affiliations:** ^1^ Jiangsu Key Laboratory of Marine Bioresources and Environment/Jiangsu Key Laboratory of Marine Biotechnology, Jiangsu Ocean University, Lianyungang, China; ^2^ Co-Innovation Center of Jiangsu Marine Bio-industry Technology, Jiangsu Ocean University, Lianyungang, China

**Keywords:** *Aeromonas* salmonicida, furunculosis, DNAzyme, SELEX, carboxyl-functionalized graphene

## Abstract

Furunculosis, which is caused by *Aeromonas salmonicida*, can induce septicemia, leading to the rapid death of fishes belonging to Salmonidae, Cyprinidae, and Fuscheridae, and lamprey. Targeting *A. salmonicida,* five DNAzyme sequences with the highest enrichment rates were selected through the Systematic Evolution of Ligands by Exponential Enrichment (SELEX). The enrichment rates were 34.78, 23.60, 8.91, 2.89, and 2.34%, respectively. The DNAzyme with the highest activity, named D-AS-2, showed specificity and sensitivity. D-AS-2 was combined with carboxyl-functionalized graphene to construct a biosensor, which showed good fluorescence response to scabies lesion samples. The diagnostic procedure was completed in <2 min and can be used for the on-site diagnosis of fish diseases. A low-cost, rapid, simple, and highly specific biosensor for the diagnosis of furunculosis was established based on DNAzyme and carboxyl-functionalized graphene.

## Introduction

With the rapid development of the mariculture industry, aquatic product output has gradually increased over the last decade (Ji et al., 2021). However, aquatic animals have a relatively simple living environment, strong dependence on human intervention, and weak resistance to environmental changes (Wang et al., 2018). In addition, aquatic animals are threatened by bacteria, viruses, or other pathogenic microorganisms. Many types of pathogenic bacteria are present in seawater, and common pathogenic bacteria include *Pseudomonas*, *Aeromonas*, and *Vibrio* (Cao et al., 2019; Rajapaksha et al., 2019).

*Aeromonas salmonicida*, a Gram-negative short *bacillus*, is among the most sensitive pathogenic bacteria in fish (Austin, 2005). *A. salmonicides* is a representative species of the thermophilic motility group, which causes furunculosis in salmon, perch, crucian carp, carp and plaice (Aakre et al., 1994; Austin et al., 2007; Jun-Chao et al., 2009; Lian et al., 2020). Acute furunculosis can lead to septicemia, and death usually occurs within 2–3 days after infection (Schulz et al., 2020). While traditional detection methods have made great contributions to the prevention and detection of pathogenic bacteria, they have many disadvantages. For example, the bacterial culturing method is time-consuming, serological tests are extremely susceptible to environmental impact, and Enzyme-linked immunosorbent assay (ELISA) is not sensitive enough and prone to antigen cross reaction (Ma et al., 2021).A simple and efficient detection method has attracted people’s attention.

DNAzymes have two parts. One is the substrate chain composed of two sequences of deoxyribonucleic acid connected by an adenine ribonucleic acid. Another is the binding target chain, which is a deoxyribonucleic acid sequence that can recognize targets. The enzyme chain of DNAzymes are obtained using Systematic Evolution of Ligands by Exponential Enrichment (SELEX) techniques (Gopinath, 2007; Teng et al., 2016)^,^. DNAzyme can specifically recognize metal ions, small molecules and proteins, such as Pb^2+^ (Liang et al., 2016), Cu^2+^ (Chen et al., 2016), Uo_2_^2+^ (Manochehry et al., 2018), histidine (He et al., 2015), lipopolysaccharide (Miao et al., 2018), glucose (Yang et al., 2015) and so on. DNAzymes are characterized by low cost, high sensitivity, strong compatibility, and ability to undergo modifications easily (Liu et al., 2017; Mcghee et al., 2017; Peng et al., 2018; Huang et al., 2020). DNAzymes are used as molecular recognition elements, and fluorescence or color change is used as a characterization signal (Cao et al., 2019). Graphene has a large surface area, can be easily functionalized, protects DNA sequences, and can quench fluorescence of aromatic molecules (Lee et al., 2008; Kim et al., 2015; Liu et al., 2018). Liu and Tian developed a biosensor targeted at *E. coli* and *Vibrio alginolyticus* through covalent self-assembly of a DNAzyme on the graphene surface (Liu et al., 2018; Tian et al., 2022). The carboxyl-functionalized graphene (CFGR) has higher dispersion ability in water than graphene. Furthermore, it has more -OH and -COOH binding sites on the surface (Liu et al., 2012). Amino (-NH_2)_-modified DNAzymes could be fixed to the surface of CFGR through an amidation reaction (Cheng et al., 2017).

**SCHEME 1:**
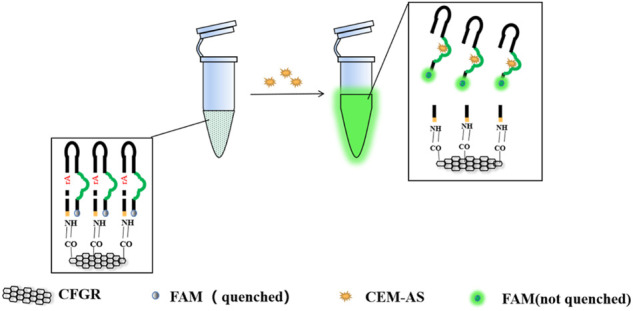
Screening principle of DNAzyme and the production method of CFGR–DNAzyme sensor.

As shown in Scheme 1, DNAzymes that could specifically recognize *A. salmonicida* were screened through SELEX (Fan et al., 2021). A biosensor combining CFGR and DNAzyme was designed wherein fluorescence was used as a signal element. This sensor has the characteristics of quick response, low cost, easy preservation, and simple operation.

## Materials and methods

### Chemicals and bacterial strains

DNA sequences for *in vitro* selection and streptavidin-coated magnetic particles were purchased from Sangon Biotech (Shanghai, China) (Table 1). *V. vulnificus*, *V. alginolyticus*, *Pseudomonas aeruginosa*, *Edwardsiella tarda, E. coli*, *Bacillus subtilis*, and *Staphylococcus aureus* were purchased from the China Center of Industrial Culture Collection*.* Ammonium persulfate, tetramethylenediamine, 2-(N-morpholine) ethanesulfonic acid (MES), and 4-hydroxyethyl piperazine ethanesulfonic acid (HEPES) were purchased from Aladdin Company(Shanghai, China). Peptone, yeast powder from XOXID (Hampshire, England) Uniq-10 Column PAGE adhesive Recovery Kit, streptomycin, magnetic beads coated with the pigment, potassium ferricyanide, and 6-mercaptoethanol were purchased from Sangon Biotech (Shanghai, China). Urea and agar were purchased from Sinopharm Chemical Reagent Co., Ltd., China. Gel-loaded dyes (6×) were purchased from Biolabs, United States. Taq DNA polymerase, 30% Acryl/Bis solution (29:1), 10× polymerase chain reaction (PCR) buffer, dNTP mixture, and DNA marker were purchased from Sangon Biotech (Shanghai, China). EDC (1-ethyl-3-(3-dimethylaminopropyl)carbodiimide hydrochloride) was purchased from Thermo, USA. NHS(N-Hydroxysuccinimide) was purchased from Shanghai Yuanye, China. CFGR was purchased from XFNANO(Nanjing, China). The random DNA library, primers, and 3′,6′-dihydroxy-3-oxospiro-isobenzofuran (FAM)-labeled substrates were all purchased from Shanghai Sangon and purified through 10% denatured polyacrylamide gel electrophoresis (dPAGE). Perch is bought from Lianyungang aquatic market, China. All bacteria were purchased from China Center of Industrial Culture Collection (CICC).*Aeromonas* Agar Base (RYAN) was purchased from Thermo Scientific™.All solutions are prepared with ultrapure water.

**TABLE 1:** Library and primer sequences.

Name	Oligonucleotide sequences (5′-3′)
Lib-N40-pool (binding target chain)	CTG​GAC​GAT​GTA​GGA​CGC​TGC​TTT​TCCAGCGT-N40-CATCGTCCAGGTCAGTGTAT
FP	CTGGACGATGTAGGACGCTGCTTTTC
RP	ATA​CAC​TGA​CCT​GGA​CGA​TG
Substrate	Biotin-ATACACTGACCTGGACGATGTrAGGACGCTGCTTTTC
K1	CAC​TAC​GGG​TCT​TGC​CAA​GCT​GCA​CCA​CGG​GTC​TTG​CCG​ATC​TG
K2	CTC​ACC​GCT​TTT​CAC​CAG​CAC​GTT​CGC​CGC​TTT​TGA​CCA​GCA​CG
D-AS-1-Q	GAA​AAG​CAG​CGT​TGG​GTA​AGG​CAG​GTT​GGT​GCG​ATG​GAC​ATG​CAA​CGT​AAC​GCA​TCG​TCC​AGG​TCA​GTG​TAT-BHQ1
D-AS-2-Q	GAA​AAG​CAG​CGT​GGG​GTA​GGA​AGG​TTG​TGT​TGA​TTG​GAA​ATG​TAG​CGA​GGC​TCA​TCG​TCC​AGG​TCA​GTG​TAT-BHQ1
D-AS-3-Q	GAA​AAG​CAG​CGT​CGA​GCA​GAG​CGA​GTA​GCT​TGA​AAA​CAT​CGG​CAT​CCA​TCG​TCC​AGG​TCA​GTG​TAT-BHQ1
D-AS-4-Q	GAA​AAG​CAG​CGT​AGG​AAG​GAA​GAA​TGA​GTG​ATA​CGA​TGA​GGA​GCA​CGT​GGC​CCA​TCG​TCC​AGG​TCA​GTG​TAT-BHQ1
D-AS-5-Q	GAA​AAG​CAG​CGT​TTG​GAG​CTC​TGC​GAC​AAA​TAG​CTC​GAA​AAC​AAG​GAT​CTG​CCA​TCG​TCC​AGG​TCA​GTG​TAT-BHQ1
Sub-FAM	FAM-ATACACTGACCTGGACGATGTrAGGACGCTGCTTTTC
Sub-NH_2_	NH_2_-ATACACTGACCTGGACGATGTrAGGACGCTGCTTTTC
D-AS-2-FAM	GAAAAGCAGCGTGGGGTAGGAAGGTTGTGTTGATTGGAAATGTAGCGAGGCTCATCGTCCAGGTCAGTGTAT-FAM

### Bacterial culture and preparation of extracellular products

*A. salmonicida* was inoculated 2% (v/v) into Nutrient gravy liquid medium (Peptone 5.0 g/L, beef extract 10 g/L, yeast extract 5.0 g/L, glucose 5.0 g/L, NaCl 5.0 g/L) and cultured at 27°C, 100 rpm for overnight. The optical density (OD) of the broth was detected at 600 nm, and culturing was ceased when the OD_600_ reached close to 1. All the culture medium was evenly divided into 1.5-ml sterile Eppendorf (EP) tubes and centrifuged at 11,000 *g* at room temperature for 5 min. The culture after removing the bacteria is called crude extracellular mixture (CEM). The supernatant was collected and stored in a refrigerator at −20°C for later use. The other strains were cultured under their optimal growth conditions, and the CEMs were prepared using the same aforementioned method.

### Purification and recovery of libraries and primers

Prior to SELEX, all initial DNA libraries and related primers were purified to avoid interference from non-target sequences. First, 100 μM lib-N40-pool, FP, and RP were taken into three sterile centrifuge tubes. Then, 10 μL of 1× urea stop solution was added to these tubes, mixed, shaken, and slightly centrifuged. The mixture was subjected to 10% dPAGE at 300 V for 30 min. The purification results were detected using the Gel Doc™ EZ Gel imaging analysis system. The target fragment was cut, recovered under an ultraviolet lamp, and dissolved in DNA extraction solution for 30 min. Then, the linked DNA was recovered through Alcohol Precipitation.

### Preparation of initial library

The random library lib-N40-pool and primers were PCR amplified according to the following scheme for preparing the initial DNA library that meets the requirements of RNA cleavage. The 50 μL PCR system included 1 μL lib-N40-pool, 2 μL primers (FP and RP), 1.5 μL of 10 mM dNTP mixture, 5 μL of 10× PCR premix, 1 μL of 5 U/μL Taq DNA polymerase, 3 μL of 25 mM MgCl_2_, and 35 μL pure water. PCR amplification conditions were as follows: pre-denaturation at 95°C for 10 min, denaturation at 95°C for 30 s, annealing at 57.5°C for 30 s, and extension at 72°C for 1.5 min.

### DNAzyme screening

Before the first round of selection, 50 μL streptomycin-coated magnetic beads (diameter: 0.5 μm) at a concentration of 50 mg/ml were placed in a 1.5-ml sterile EP tube. The beads were washed three times with 500 μL Buffer A (10 mM Tris-HCl, 1 mM EDTA, 1 M NaCl, and 0.02% Tween-20). Then, 500 μL of PCR-purified product dissolved with Buffer A (containing biotin label) was mixed with the cleaned magnetic beads (concentration of magnetic beads: 5 mg/ml). The reaction tube was placed on a DNA mixer and incubated at 37°C for 30 min. After the reaction was completed, unbound DNA (supernatant) was removed using a magnetic separator, and the beads were cleaned twice with 500 μL Buffer A. Then, 500 μL of 0.2 M NaOH was added to the reactant mixture and mixed well. The mixture was allowed to stand at room temperature for 2 min and then repeatedly rinsed to ensure the pH is maintained at 7.0. Then, 150 μL CEM of *A. salmonicida* and equal volume of Buffer B (100 mM HEPES, 300 mM NaCl, 30 mM MgCl_2_, and 0.02% Tween-20, pH 7.5) were added to the mixture and mixed evenly. The samples were placed on a DNA-mixing device and reacted for 1 h at 37°C. Finally, the fragments were obtained through magnetic separation and recovered through alcohol precipitation. The initial length was restored through PCR. After each round of selection, the required library was prepared using the magnetic bead method-PCR. The CEMs of *V. vulnificus*, *V. alginolyticus*, *P. aeruginosa*, *Edwardsiella tarda*, *E. coli*, *B. subtilis,* and *S. aureus* were used in the reverse selection.

### High-throughput sequencing

After nine rounds of selection, the sequences were enriched with alcohol, precipitated, dried, and dissolved in 100 μL ultrapure water. The sample was packaged and sent to Shanghai Sangon for high-throughput sequencing.

### Screening active DNAzyme and specific detection

The five candidate DNAzyme sequences with the highest enrichment rates were synthesized by Shanghai Sangon. The 3′ end of the binding target chain was modified with a quencher, and substrates were modified with FAM at the 5′ end. A total of 7.5 µM binding target chain was added to 5 µM substrate (diluted with ultrapure water) in a light-resistant tube, heated at 95°C for 3 min, and cooled naturally to room temperature to allow them combine into a DNAzyme. Fluorescence signals were monitored using polyacrylamide gel and kinetic methods to demonstrate pyrolysis reactions. A total of 10 µL CEM-AS was mixed with 20 μL of 2× Buffer B, 16 μL ultrapure water, and 4 μL DNAzyme, and the mixture was incubated for 30 min. The reaction was terminated by adding 10 μL of 2× dye (containing 8 M urea) on the gel. The samples were subjected to 15% dPAGE and separated through electrophoresis at 150 V for 50 min. Results were analyzed using the Bio-RAD GelDocTM EZ imaging system (Bio‐RAD, USA). To measure DNAzyme activity, 41 μL ultrapure water, 44 μL Buffer B, 4 μL DNAzyme, and 10 μL CEM-AS was mixed. Using a microplate analyzer, fluorescence intensity was measured for 2 h at excitation and emission wavelengths of 490 and 520 nm, respectively. To determine the specificity of DNAzymes, 10 ml CEM-AS was replaced by CEM-VA, CEM-VV, CEM-PA, CEM-ET, CEM-EC, CEM-BS, and CEM-SA. Experiments were performed using the optimum DNAzyme tested above, and fluorescence signals were monitored using polyacrylamide gel and kinetic methods to demonstrate cleavage reactions.

### Optimization of detection conditions

The optimized pH range was 4.5–10.0; MES was used for the buffer at pH 4.5, 5, 5.5, 6, and 6.5; HEPES was used attain pH 7 and 8; and NaOH was used to attain pH 8.5–10.0. Instead of Buffer B, the fluorescence signal was monitored using the aforementioned kinetic method. Then, DNAzymes with the highest enzyme activity were selected to adjust the ratio of the substrate to the binding target chain. The fluorescence signal was monitored to determine the optimal concentration of the substrate and binding target chain.

Different monovalent ion buffer solutions (45 μL) were prepared by combining five monovalent metal ions (Rb^+^, Cs^+^, K^+^, Li^+^, and Na^+^, final concentration of 300 mM) and 100 mM HEPES. The pH of these solutions was adjusted with 41 µL ultrapure water. After the mixture of 4 μL DNAzyme and 10 μL CEM-AS was uniform, fluorescence values within 1 h were read using a microreader. Then, different divalent metal ion solutions (Co^2+^, Fe^2+^, Zn^2+^, Mg^2+^, Ba^2+^, Mn^2+^, Ca^2+^, and Sr^2+^, with a final concentration of 30 mM) were prepared by combining the optimal metal ions. The fluorescence was monitored to determine the optimal divalent ions.

### CEM-AS identification

CEM-AS was mixed with proteinase K, papain, pepsin, *β*-mercaptoethanol, and parenzyme at a 1:1 ratio and incubated at the optimum temperature of the enzymes for 1 h. Then, the DNAzyme activity was detected by monitoring the fluorescence.

### Preparation of the CFGR–DNAzyme sensor

The homogenized CFGR solution was obtained through ultrasonic treatment of 1 mg/ml CFGR for 6 h. The 5′ end of the substrate was modified with -NH_2_, and 3′ end of the binding target chain was modified with FAM by Shanghai Sangon. The DNAzyme (diluted with ddH_2_O) was incubated at 95°C for 3 min and cooled at room temperature to achieve a final concentration of 3 μM. Then, 20 μL CFGR solution, 20 μL Buffer B, 10 μL of 3 μM DNAzyme, and an aqueous solution containing 0.4 M EDC and 0.1 M NHS was added to 200 μL of DNAzyme in the EP tube. The EP tube was then placed in a 37°C constant temperature incubator for 12 h.

### Optimization of the CFGR–DNAzyme sensor

First, 20 μL CFGR solution, 20 μL Buffer B, 10 μL of 3 μM DNAzyme solution, and aqueous solution containing 0.4 M EDC and 0.1 M NHS were added to a 96-well plate. In the fluorimeter, the fluorescence value was recorded every 10 min, and recording was stopped when the fluorescence no longer changed.

Then, 10, 15, 20, 25, and 30 μL of 1 mg/ml CFGR solution were added to the EP tube. This was followed by the addition of 20 μL Buffer B, 10 μL of 3 μM DNAzyme solution, and 5 μL aqueous solution containing 0.4 M EDC and 0.1 M NHS. The EP tube was placed in a 37°C constant temperature incubator for 12 h. Then, 40 μL CEM-AS and ultrapure water were added to the experimental and control groups, respectively. The changes in fluorescence were observed on the Blue-Light Transilluminator (Safe Imager ™) and photographed. Meanwhile, the specific values were recorded using the fluorescence microplate reader.

Different concentrations of DNAzyme (400, 500, 600, 700, and 800 nM) were added to the EP tubes, and then the most appropriate amount of CFGR was added, followed by the addition of 20 μL Buffer B and 5 μL aqueous solution containing 0.4 M EDC and 0.1 M NHS. Then, 40 μL CEM-AS and ultrapure water were added to the experimental and control groups, respectively.

The changes in fluorescence were observed on the Blue-Light Transilluminator and photographed. Meanwhile, the specific values were recorded using the fluorescence microplate reader.

After adding 10, 15, 20, 25, and 30 μL Buffer B into the EP tube, the most appropriate amounts of CFGR and DNAzyme, and 5 μL aqueous solution containing 0.4 M EDC and 0.1 M NHS were added. Then, 40 μL CEM-AS and ultrapure water were added to the experimental and control groups, respectively.

The changes in fluorescence were observed on the Blue-Light Transilluminator and photographed. Meanwhile, the specific values were recorded using the fluorescence microplate reader.

### Specificity of the CFGR–DNAzyme sensor

The CFGR–DNAzyme sensor was prepared by adding 40 μL CEM-AS, CEM-VA, CEM-VV, CEM-PA, CEM-ET, CEM-EC, CEM-BS, and CEM-SA. The fluorescence was observed on the Blue-Light Transilluminator and photographed. The specific values were recorded using the fluorescence microplate reader.

### Pathogenic experiment of perch

Healthy California perch were raised in a 20-L fresh water tank with three fish in each group. During the experiment, the tank was continuously aerated at 20°C, and the water was changed each day. After culturing the bacteria at 27°C for 14 h, the bacterial concentration was determined using the McFarland turbidimetric method (Kolmert et al., 2000). Then, 100 μL bacterial solution was injected into the cavum abdominis of fish in the experimental group, and 100 μL sterile saline was injected into the control group. The fish were observed at regular intervals, and lesions of the dying perch were collected for grinding, centrifugation, and ultrafiltration for subsequent experiments. The crushed liver of the perch was centrifuged in a gradient and then cultured with *Aeromonas* Medium Base (Ryan) at 27°C. The isolated bacteria were sent to Shanghai Sangon for 16S rDNA sequencing analysis. The phylogenetic tree was constructed using MEGA version 8.

### Sensor application

The obtained focal fluid was added to the developed sensor, and whether the focal sample was cleaved by the DNAzyme was determined using the fluorescence and 15% polyacrylamide gel methods. At the same time, genomes from liver and pancreas lapping solutions were extracted from the control and sample groups using Bacteria Genomic DNA Kit. The *exe* D gene was amplified by primers K1 and K2. (Karlyshev and Macintyre, 1995).

### Sensor performance testing

At the same DNAzyme concentration, the quencher group, GO, and CFGR were used to quench the fluorescence, and the initial fluorescence of the three was compared with the fluorescence after CEM-AS addition. To verify the stability of the DNAzyme–CFGR sensor, the sensor was placed in an environment of 37°C to detect changes in its fluorescence.

## Results

### DNAzyme selection

**FIGURE 1:**
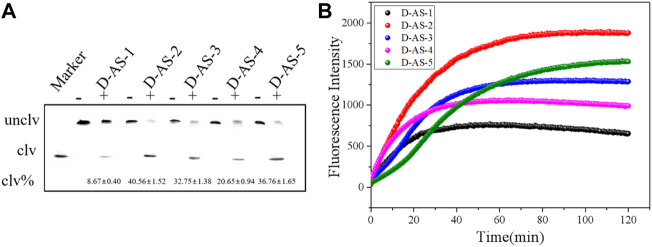
**(A)** 15% dPAGE analysis of candidate sequence activity within 30 min Unclv: Complete DNAzyme sequence; clv: pyrolysis production.clv%:percentage of pyrolysis production; binding chain7.5 μM and substrate 5 μM. **(B)** Fluorescence assay to determine the activity of candidate sequences.

Nine rounds of SELEX were conducted, and anti-screening targets were included in the second, fourth, sixth, and eighth rounds to improve the specificity of DNAzymes. The DNA library containing 40 random bases was used to separate active and inactive sequences, and the positive and negative screening processes were cross-used. The DNA obtained in the ninth round was extensively sequenced, and 1211 original sequences were obtained (Supplementary Table S1). The number of repeats of different sequences was calculated, and the maximum enrichment degree was attained (34.78%), reaching the expected enrichment target. The first five candidate sequences with high enrichment were named D-AS-1, D-AS-2, D-AS-3, D-AS-4, and D-AS-5 respectively. These first five sequences (Table 2) were chemically synthesized and modified with quencher groups at the 3′ end, and the 5′ end of the substrate DNA chain was modified with FAM tags. When the two chains were complementary and paired, the fluorescence group was close to the quencher group, and the fluorescence was quenched. Its cleavage activity was evaluated in the presence of CEM-AS. As shown in Figures 1A,B, DNAzyme can catalyze the cleavage of RNA embedded in the DNA substrate in the presence of CEM-AS, as detected by 15% polyacrylamide gel and fluorescence methods, and the DNAzyme called D-AS-2 exhibited the highest activity. Therefore, we selected D-AS-2 candidate sequences for further experiments.

**TABLE 2:** Candidate sequences.

Top 5	Sequences of the random region	Percentageof total sequences
D-AS-1	GAAAAGCAGCGTTGGGTAAGGCAGGTTGGTGCGATGGACATGCAACGTAACGCATCGTCCAGGTCAGTGTAT	34.78%
D-AS-2	GAAAAGCAGCGTGGGGTAGGAAGGTTGTGTTGATTGGAAATGTAGCGAGGCTCATCGTCCAGGTCAGTGTAT	23.60%
D-AS-3	GAAAAGCAGCGTCGAGCAGAGCGAGTAGCTTGAAAACATCGGCATCCATCGTCCAGGTCAGTGTAT	8.91%
D-AS-4	GAAAAGCAGCGTAGGAAGGAAGAATGAGTGATACGATGAGGAGCACGTGGCCCATCGTCCAGGTCAGTGTAT	2.89%
D-AS-5	GAAAAGCAGCGTTTGGAGCTCTGCGACAAATAGCTCGAAAACAAGGATCTGCCATCGTCCAGGTCAGTGTAT	2.34%

### Optimization of reaction condition

**FIGURE 2:**
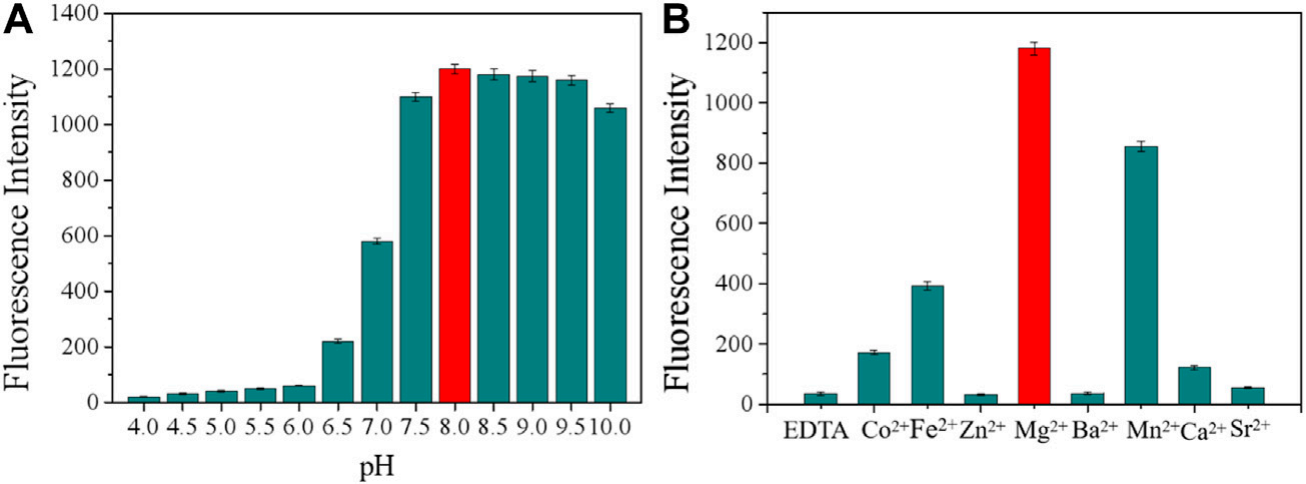
**(A)** Cleavage at different pH values. **(B)** Effect of different divalent metal ions on the cleavage activity. Addition of 300 mM EDTA fully inhibited the cleavage (all DNAzymes were formed in Buffer B).

Appropriate pH and catalytic ion species critically influence the cleavage activity, and therefore, these two determination conditions were optimized. First, we chose several gradients of 4.5, 5, 5.5, 6, 6.5, 7, 7.5, 8.0, 8.5, 9.0, 9.5, and 10.0 to optimize the pH of the buffer solution. As shown in Figure 2A, the cleavage activity of the DNAzyme was the highest at pH 8.0, and so, the buffer solution with pH 8.0 was used for subsequent experiments. EDTA can form a with divalent ions. After combining EDTA with Buffer B, EDTA can be used as a buffer reagent in fluorescence detection, as shown in Figure 2B. EDTA-treated Buffer B exhibited no obvious cleavage activity, and so, divalent metal ions have a crucial influence on the cleavage activity. When the common monovalent ion Na^+^ was considered as the only monovalent ion, Mg^2+^ showed high cleavage activity after different divalent ions were added, and Mg^2+^ will be used for subsequent experiments.

### DNAzyme specificity analysis

**FIGURE 3:**
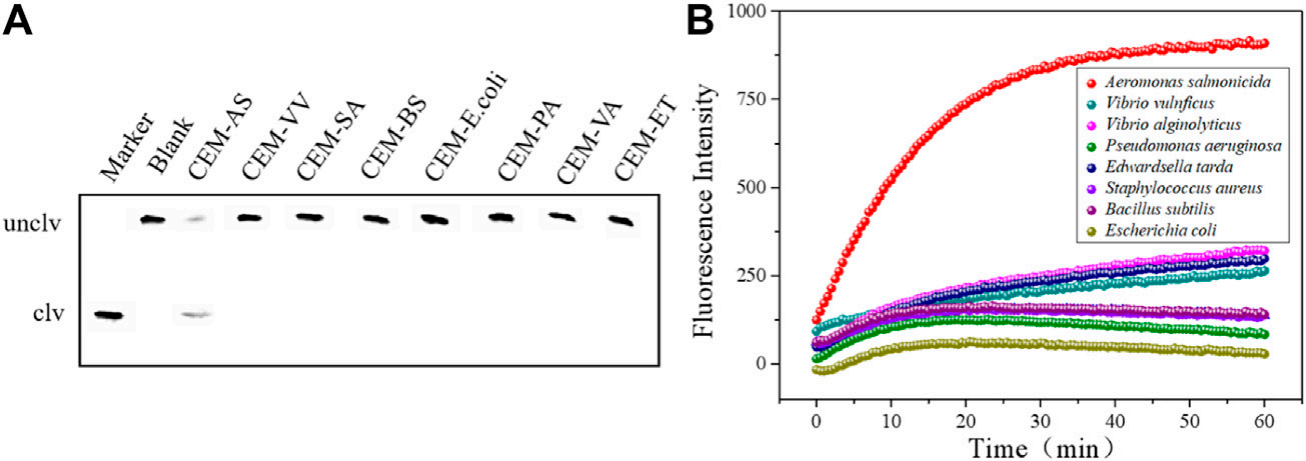
**(A)** dPAGE analysis of D-AS-2 specificity detection within 30 min. **(B)** Fluorescence assay of D-AS-2 to seven pathogenic bacteria.

Several common pathogenic bacteria were selected as detection samples, as shown in Figures 3A,B. In the presence of CEM-AS, DNAzyme was cut and fluorescence changes were obvious. When the target was other pathogenic bacteria, DNAzyme could not be cut and fluorescence did not change. Therefore, D-AS-2 has good specificity and can be used in sensor production.

### DNAzyme target identifications

**FIGURE 4:**
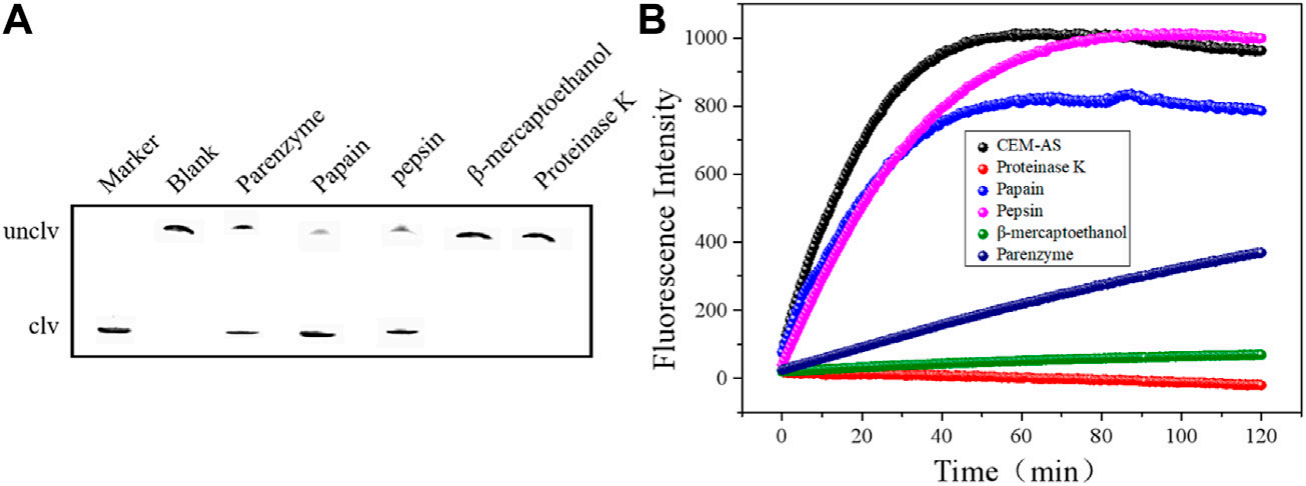
**(A)** 15% dPAGE analysis of various enzymes (proteinase K, papain, pepsin, *β*-mercaptoethanol, and parenzyme) treated with D-AS-2. **(B)** Fluorescence assay for determining D-AS-2 activity.

After the treatment of CEM-AS with proteinase K, papain, pepsin, *β*-mercaptoethanol, and parenzyme, the proteins in CEM-AS are destroyed. As shown in Figure 4, after CEM-AS was treated with proteinase K and *β*-mercaptoethanol, D-AS-2 showed no obvious cleavage activity. Papain and pepsin had little effect on the cleavage activity. After parenzyme treatment, D-AS-2 cleavage activity decreased significantly. Proteinase K is a strong proteolytic enzyme, *β*-mercaptoethanol can destroy the fourth or third order structure of proteins, papain and pepsin can only break down part of the peptide bonds in proteins, and parenzyme, the most specific protease, is a serine proteolytic enzyme. Therefore, D-AS-2 is speculated to target the protein and contains part of the serine protein.

### Detection of the CFGR–DNAzyme sensor

**FIGURE 5:**
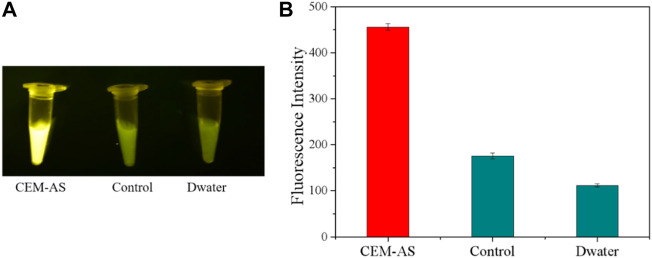
**(A)** Fluorescence response of the DNAzyme–CFGR sensor. **(B)** Fluorescence assay to determine the feasibility of the DNAzyme–CFGR sensor. (Control: add liquid medium CM0841 without bacteria).

The selected D-AS-2 was used for biosensor production. First, CFGR was added to the EP tube as a carrier of DNAzyme, and CFGR could quench the FAM fluorescence label on D-AS-2. EDC and NHS were added to activate the carboxyl group of CFGR, and finally, Buffer B was added. When EP tubes were incubated at 37°C, -NH_2_ of D-AS-2 could bind to the carboxyl group of CFGR. As shown in Figures 5A,B, no fluorescence change occurred when ultrapure water was added into the sensor. However, a weak fluorescence change was observed after the addition of the medium, which was due to the darker color of the medium itself, and a significant fluorescence change occurred after the addition of CEM-AS.

### Optimization of detection conditions

**FIGURE 6:**
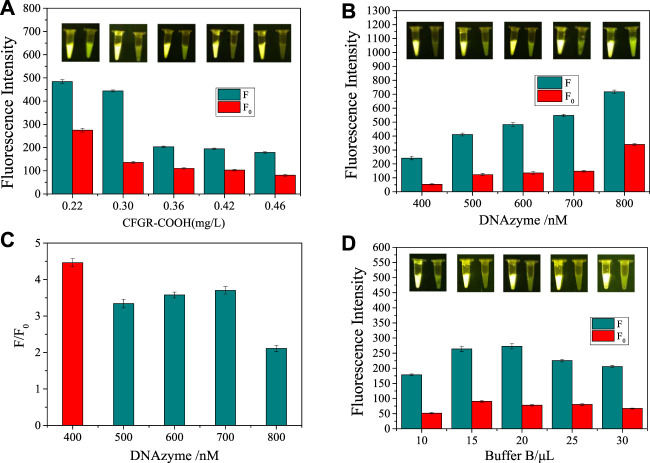
**(A)** Optimization of CFGR and DNAzyme binding time. **(B)** Optimization of DNAzyme content. **(C)** The ratio of F to F_0_. **(D)** Optimization of Buffer B content. (F: The final value of the fluorescence intensity, F_0_: The initial value of the fluorescence intensity, The insets of **(A, B, D)** show the real-time fluorescence changes in the sensor).

The appropriate binding time, the amount of CFGR, the concentration of DNAzyme, and the amount of Buffer B all play a crucial role in detection by the sensor. Therefore, different measurement conditions were optimized. First, the binding time of DNAzyme and CFGR was optimized. As observed in Figure 6A, fluorescence decreased to the lowest value. The final concentrations of CFGR were 0.22,0.30,0.36,0.42 and 0.46 mg/L. As shown in Figure 6B, 0.30 mg/L was the optimal CFGR supplemental level. Background fluorescence plays a large interference role for the sensor. To obtain a higher detection efficiency, the DNAzyme concentration is optimized to find a balance between the obvious fluorescence signal and the detection signal. As shown in Figure 6C, when the DNAzyme concentration is 400 nM, the ratio of the fluorescence signal with CEM-AS to the blank group is the largest, and the amount of DNAzyme added was small. Therefore, the DNAzyme concentration was selected as 400 nM for subsequent experiments. Finally, several gradients of 10, 15, 20, 25, and 30 μL were selected to optimize the amount of Buffer B. As observed in Figure 6D, the fluorescence signal was most obvious when the amount of Buffer B was 20 μL.

### Sensitivity and specificity of the sensor

**FIGURE 7:**
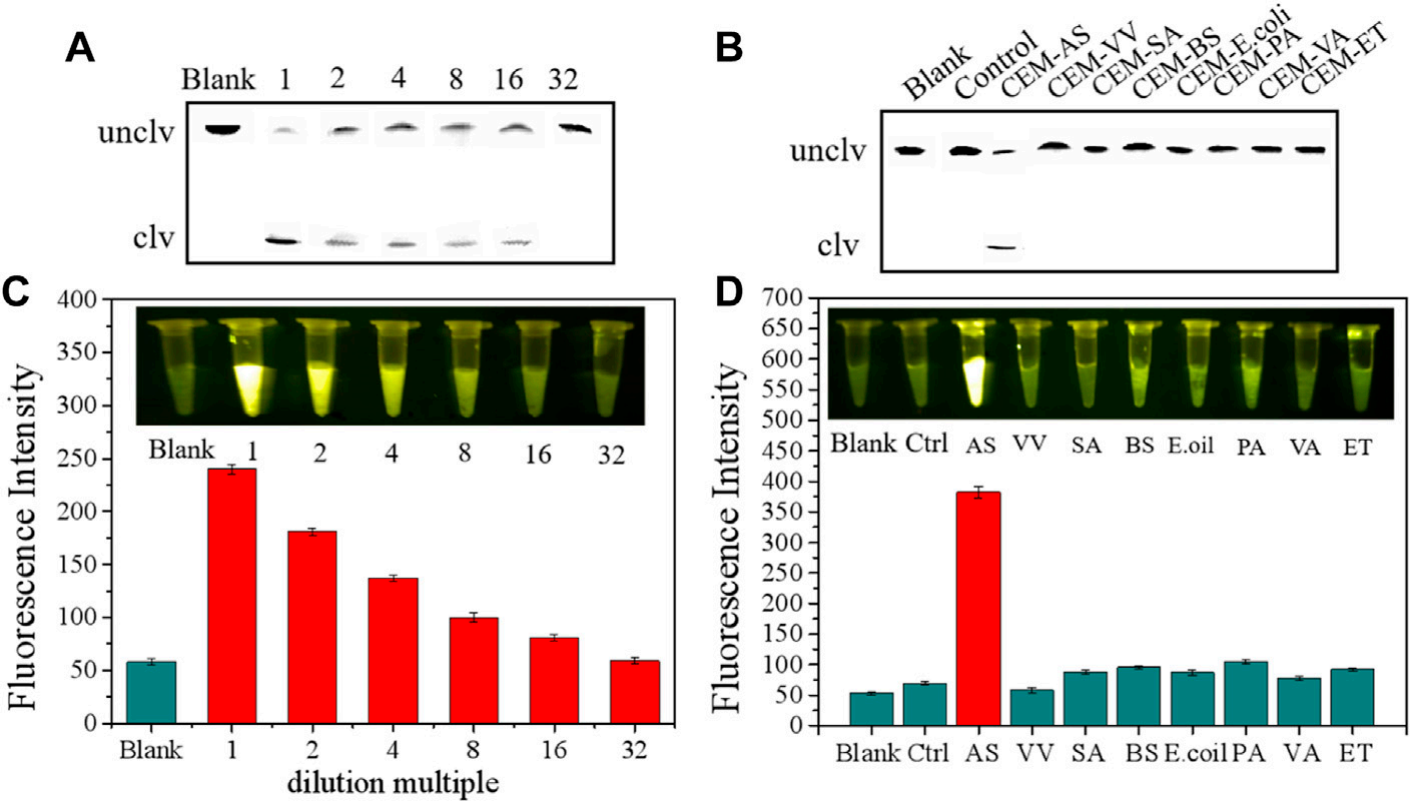
**(A)** dPAGE Analysis of sensor detection limit **(B)** dPAGE analysis of sensor sensitivity. **(C)** Fluorescence assay to determine the detection limit of the sensor. **(D)** Fluorescence assay to determine the sensitivity of sensor.

Before performing sensitivity and specificity tests, we conducted some preparatory work. On the one hand, the culture medium of *A. salmonicida* (1.5 × 10^9^ CFU/ml) was diluted to test sensitivity. On the other hand, we prepared extracellular products by using bacteria employed in reverse screening for specificity tests. Sensitivity was measured through sensor and fluorescence methods and was further verified using the polyacrylamide method. As can be seen from Figures 7A,B, the extracellular products of *A. salmonicida* culture medium (1.5 × 10^9^ CFU/ml), which were diluted 16 times, were detected by the sensor. A good sensor should only work on one target and not on multiple targets. As shown in Figure 7B, the sensor showed a strong fluorescence signal only in the presence of CEM-AS, whereas the extracellular products from other bacteria could not cause fluorescence changes, indicating that the sensor had excellent detection specificity. Combined with the sensitivity of the bacteria, the sensor could be used for diagnosing fish diseases during breeding.

### Application on the infected perch

**FIGURE 8:**
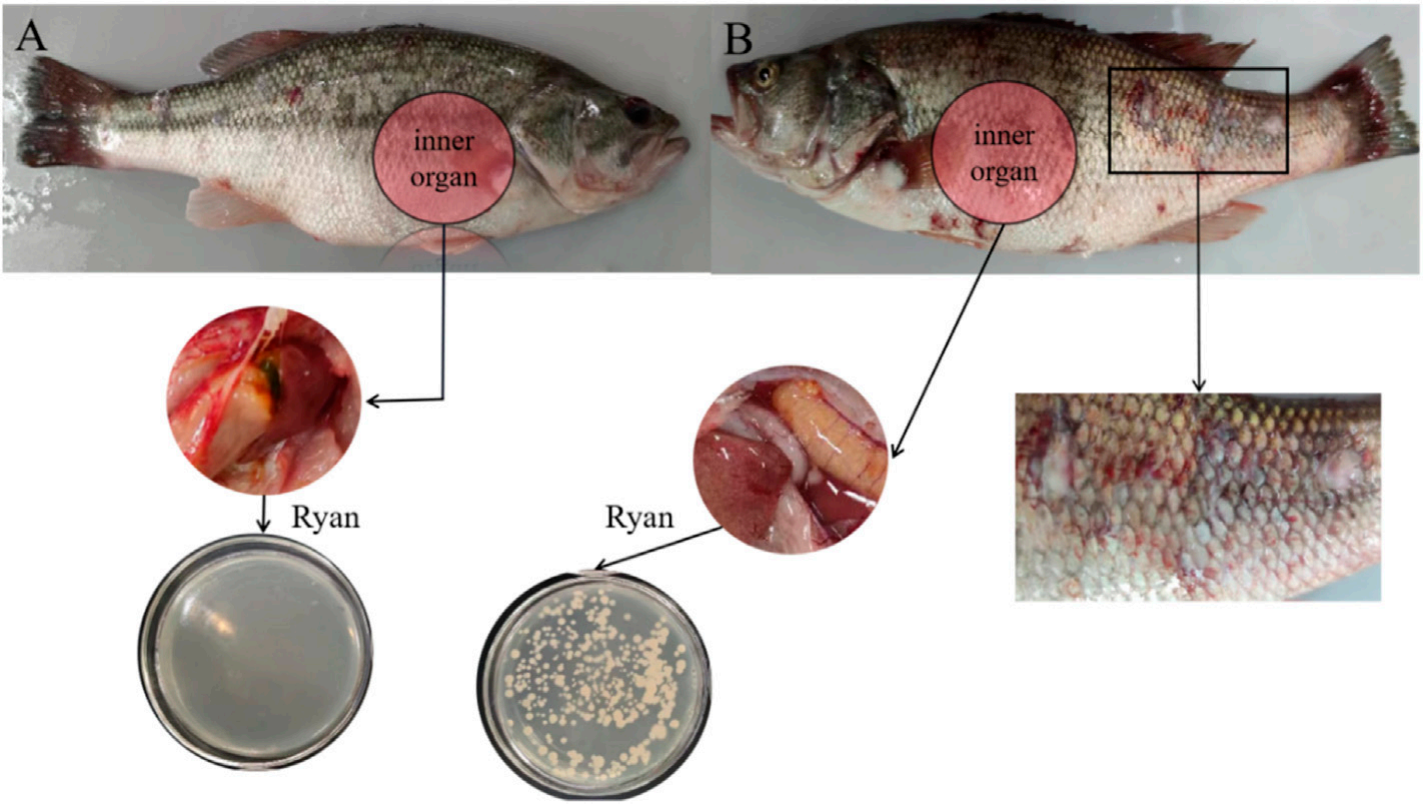
**(A)** The body surface and inner organ of a perch injected with normal saline. **(B)** The body surface and inner organ of a perch injected with CEM-AS.

After 48 h, the experimental perch group began to die. After 50 h, all three perch died. At this time, the control perch group had a normal activity and no disease. As can be seen through comparison of Figures 8A,B, many wounds were observed on the surface of the fish body infected by bacteria, including pus and blood exudate, bleeding from the base of the pelvic and caudal fins, and swelling of muscles. The abdominal anatomy of the bacteria-infected fish was determined. A comparison of the inner organ revealed blood ascites in the fish abdomen, swelling of the fish liver and pancreas, and loss of blood.

### Sensor application experiment

**FIGURE 9:**
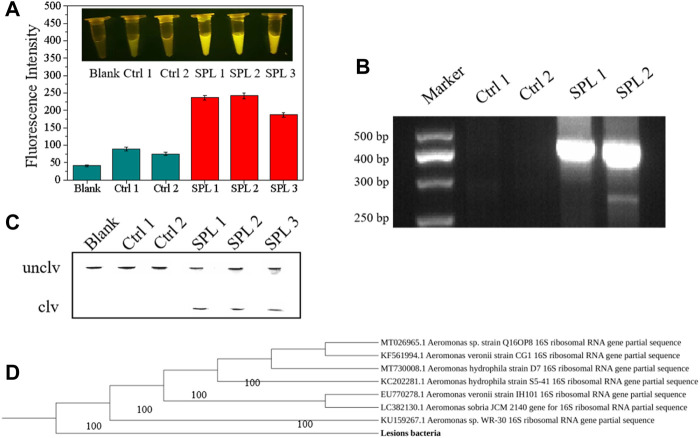
**(A)** Fluorescence response of the DNAzyme–CFGR sensor to fish samples. **(B)** dPAGE analysis of fish samples. **(C)** Agarose gel diagram of *exe* D gene after amplification **(D)** Phylogenetic tree of focal bacteria. (Ctrl 1: Liver of an uninfected fish, Ctrl 2: Pancreas of an uninfected fish, SPL 1:The Ascites of an infected fish, SPL 2:The Liver of an infected fish, SPL 3:The pancreas of an infected fish).

To evaluate the performance of the DNAzyme–CFGR sensor, samples of lesions obtained from the perch were used for fluorescence detection. As can be seen from Figure 9A, ascites, liver, and pancreas samples of bacteria-infected fish all have high fluorescence values, and the fluorescence changes can be clearly seen through naked eyes, whereas liver and pancreas samples from the control group showed no obvious fluorescence changes. *A. salmonicida* causes fish furunculosis, mainly due to the secreted a-layer protein and extracellular protease (Frey and Origgi, 2016). A certain number of target proteins are present in the diseased fish, which will cause the specific cleavage reaction of the sensor. As shown in Figure 9C, the lesion samples of infected fish can cause the reaction of the DNAzyme. Ascites could be used as a sample for furunculosis because they are readily available and relatively clear. After 16S rRNA sequencing and phylogenetic tree establishment (Figure 9D, bacteria isolated from the liver of the diseased fish were found to be *Aeromonas* sp. *Exe* D gene is the proven virulence gene of *A. salmonicides*, and based on the primers, the sequences are 421 bp. As the Figure 9B shows that the liver and pancreas samples of infected bass had obvious bands; by contrast, the uninfected bass could not be detected the sequences of *exe* D. Therefore, the fish furunculosis was caused by the Pathogenic *A. salmonicides.* The DNAzyme–CFGR sensor had an obvious signal response to the fish infected with furunculosis, indicating that the sensor can indeed be used for diagnosing fish furunculosis.

### Sensor performance testing

**FIGURE 10:**
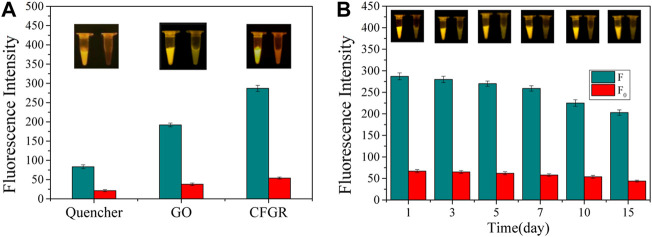
**(A)** Fluorescence intensity of the quencher group, GO, and CFGR. **(B)** The change in fluorescence intensity with days. (F: The final value of the fluorescence intensity, F_0_: The initial value of the fluorescence intensity. The insets show the real-time fluorescence changes in the sensor).

The quencher group, GO, and CFGR were respectively used as fluorescence quenching materials. Figure 10A shows that all three of them have a good fluorescence quenching effect, but the initial fluorescence value of the three (F_0_) and the fluorescence value F after the reaction are quite different. The ratios of F/F_0_ of the three were 3.90, 5.05, and 5.31, respectively. By contrast, both GO and CFGR had higher F/F_0_ ratios than the quencher group. CFGR had slightly higher F/F_0_ ratios than GO, but CFGR had the highest F value. According to the property analysis of GO and CFGR, CFGR had higher dispersion in water and more carboxyl binding sites. As observed in Figure 10B, after the sensor was prepared, it still had a good fluorescence response within 15 days at 37°C. Therefore, the DNAzyme–CFGR sensor has the characteristics of obvious fluorescence signal, fast, stable, and simple production. The sensor can be prepared into a detection kit, wherein each component can be stored at 4°C for >6 months, the preparation process is simple, and the high temperature environment during summer also has good stability. This sensor can be used as the basis for diagnosing fish furunculosis, and it generates an obvious signal response by directly using the water in the abdomen of diseased fish as the detection sample.

## Conclusion and discussion

The DNAzyme that specifically identified *A. salmonicida* was successfully selected, and a DNAzyme–CFGR biosensor for the rapid detection of *A. salmonicida* was developed. The detection time was <2 min, and the sensor showed an obvious fluorescence signal in response to the CEM of *A. salmonicida* (1 × 10^8^ CFU/ml) and the sick fish organs. The CEM was identified as a protease K-hydrolyzable protein. Therefore, the sensor could rapidly diagnose fish furunculosis on-site.

*A. salmonicida* is one of the bacteria that causes the highest incidence of fish infection globally. Therefore, a simple and convenient detection method is urgently required. DNAzyme-based sensors have been established for detecting aquatic pathogens such as *A. hydrophila*, *V. vulnificus*, *V. lysozyme,* and *V. anguillarum*. These sensors have high sensitivity and specificity, and are highly convenient. Therefore, the sensor of *A. salmonicida* based on DNAzyme has a great prospective application.

At present, the culture separation method is the traditionally used detection method. In this method, bacteria in the fish lesions are isolated using the *Aeromonas* Isolation Agar (Base) medium, and then *A. salmonicida* is identified through the complex process of physiological and biochemical identification and serological experiments (Lee et al., 2015; Váradi et al., 2017). These steps are time-consuming and cumbersome, and the process is vulnerable to contamination by other bacteria. Some new methods have been recently developed for *A. salmonicida* detection. They mainly include methods PCR using specific nucleic acid sequences in bacteria and antibody detection using a highly specific antigen and antibody combination. Ji-young Ahn (Ahn et al., 2018) constructed a surface plasmon resonance biosensor for detecting *V. parahaemolyticus* by using nucleic acid as the target, and the detection limit of the sensor was 4 × 10^8^ CFU/ml. By amplifying the V3 variable region of the 16S rRNA gene and verifying the results by using the denaturing gradient gel, Quinn (Quinn and Stevenson, 2012) et al. designed a PCR analysis technique for detecting *A. salmonicida*. On the basis of PCR technology, new variants of this technology have been developed, such as multiplex PCR, fluorescence quantitative PCR, and reverse transcription multiplex PCR. Kulkarni (Kulkarni et al., 2009) et al. designed a specific primer targeting the gyrB gene of *A. salmonicida* to establish a rapid isothermal amplification assay capable of detecting this pathogen in 45 min. An antigen–antibody-based immunoassay has been widely used in environmental monitoring, food safety, and clinical practice. Somamoto (Somamoto et al., 2018) et al. used a monoclonal antibody to detect *A. salmonicida* through immunofluorescence staining. Saleh (Saleh et al., 2011) et al. conducted a colorimetric assay of gold nanoparticles coupled with polyclonal antibodies and detected *A. salmonicida* in fish tissues within 45 min; it had a detection limit of 1 × 10^4^ CFU/ml.

The combination of DNAzyme and nanoparticles could enhance the detection of sensors. AuNPs, AgNCs and graphene have been used to combine with DNAzyme to make sensors. He (He et al., 2019) et al. developed an AuNPs-DNAzyme molecular motor biosensor that could detect detection of microRNA (miRNA)-155 rapidly and sensitively. Zhao (Zhao et al., 2022) et al. developed a AuNPs@DNAzyme senor for electrochemical detection of multiple DNA glycosylases, AuNPs as the carrier of DNAzyme, AuNPs@DNAzyme could release electrochemical signals after target identification. Zheng (Zheng et al., 2018) et al. presented a novel DNA-templated fluorescent silver nanoclusters (DNA-AgNCs) based sensing system integrated with magnetic nanoparticles (MNP)-DNAzyme-AChE complex for detection of pathogenic bacterial *E. coli.* Researches have indicated that Nanomaterials could improve the performance of DNAzymes sensors significantly.

Compared with the aforementioned methods, this method using the DNAzyme D-AS-2 has higher specificity, shorter detection time, and less dependence on professional instruments for *A. salmonicida* detection. We thus provide a new method for the rapid detection of *A. salmonicida* and for the diagnosis of furunculosis caused by this bacterium, thereby allowing the timely treatment of the disease.

## Data Availability

The datasets presented in this study can be found in online repositories. The names of the repository/repositories and accession number(s) can be found in the article/Supplementary Material.
